# Obstetric antiphospholipid syndrome carries an increased lifetime risk for obstetric and thrombotic complications—a population-based study

**DOI:** 10.1016/j.rpth.2024.102430

**Published:** 2024-04-29

**Authors:** Ariel Katherine Rhein, Anat Rabinovich, Ran Abuhasira, Shir Lubaton-Barshishat, Offer Erez

**Affiliations:** 1 The Joyce & Irving Goldman Medical School, Faculty of Health Sciences, Ben-Gurion University of the Negev, Beer-Sheva, Israel; 2 Thrombosis and Hemostasis Unit, Hematology Institute, Soroka University Medical Center and Faculty of Health Sciences, Ben-Gurion University of the Negev, Beer-Sheva, Israel; 3 Clinical Research Center, Soroka University Medical Center, Beer-Sheva, Israel; 4 Department of Obstetrics and Gynecology, Soroka University Medical Center, Beer-Sheva, Israel; 5 Department of Obstetrics and Gynecology, Hutzel Women's Hospital, Wayne State University, Detroit, Michigan, USA

**Keywords:** antiphospholipid syndrome, fetal death, preeclampsia, pregnancy outcome, thrombosis

## Abstract

**Background:**

Antiphospholipid syndrome (APS) can present with either a thromboembolic event (thrombotic APS, TAPS) or an obstetric complication (obstetric APS, OAPS). Data on long-term complications in the different APS phenotypes are limited.

**Objectives:**

We aimed to compare obstetric history, antiphospholipid antibody profiles, obstetric and thromboembolic complications, and pregnancy outcomes between TAPS and OAPS.

**Methods:**

This retrospective cohort study included women who delivered singleton pregnancies between 1998 and 2020. One hundred sixteen thousand four hundred nine women were included, resulting in 320,455 deliveries. Among the included patients, 71 were diagnosed with APS, 49 were classified as OAPS, and 22 as TAPS. The demographics, obstetric, neonatal, and thrombotic outcomes were compared among TAPS, OAPS, and the general obstetric population.

**Results:**

OAPS patients had an increased risk of thrombotic events compared with the general obstetric population (odds ratio [OR] 18.0; 95% CI, 8.7-37.2). In pregnancies following the diagnosis of APS, despite standard antithrombotic treatment, OAPS patients exhibited an elevated risk of placenta-related and neonatal complications compared with the general obstetric population (late fetal loss [adjusted OR {aOR}, 15.3; 95% CI, 0.5-27.5], stillbirth [aOR, 5.9; 95% CI, 2.2-15.4], placental abruption [aOR, 4.8; 95% CI, 1.5-15.3], preeclampsia [aOR, 4.4; 95% CI, 2.5-7.7], fetal growth restriction [aOR, 4.3; 95% CI, 8.5-27.5], small for gestational age neonate [aOR, 4.0; 95% CI, 2.4-6.6], and low Apgar scores [Apgar'1: aOR, 2.6; 95% CI, 1.3-10.4; Apgar'5: aOR, 3.7; 95% CI, 1.3-10.4]). TAPS patients exhibited increased risk of preeclampsia (aOR, 3.1; 95% CI, 1.2-8).

**Conclusion:**

OAPS patients exhibit a heightened risk of thrombotic events compared with the general obstetric population. Despite treatment, OAPS and TAPS still presented obstetric complications. These findings, after confirmation in prospective studies, need to be taken into consideration when planning the treatment approach for these patients.

## Essentials

• Thrombotic antiphospholipid syndrome (APS) and obstetric APS (OAPS) are 2 phenotypes of APS.
• This study compared data on long-term complications in the different APS phenotypes.
• OAPS patients had an increased risk of thrombotic events.
• Despite treatment, OAPS and thrombotic APS still presented obstetric complications.

## 1 Introduction

Antiphospholipid syndrome (APS) is an acquired autoimmune disorder characterized by vascular thrombosis and pregnancy complications occurring in patients with persistent elevated concentrations of antiphospholipid antibodies (aPLs) [[1], [2], [3]]. In 2013, new criteria were proposed to separate 2 different entities of APS: 1) thrombotic APS (TAPS), characterized by venous, arterial, or microvascular thrombosis, and 2) obstetric APS (OAPS), associated with obstetric complications including recurrent pregnancy loss, fetal death, stillbirth, and premature birth due to preeclampsia, eclampsia, or placental insufficiency [4]. Standard treatment for pregnant women with a previous diagnosis of APS, either of obstetric or thrombotic phenotype, is low-molecular-weight heparin (LMWH) and low-dose aspirin during gestation and 6 weeks postpartum [5]. Despite standard treatment, APS patients still develop severe obstetric and thrombotic complications [2,6]. Previous studies suggested distinct pathophysiologic mechanisms for thrombotic and obstetric phenotypes [7]. Considering the scarcity of data on the impact of APS phenotype on pregnancy outcomes, we conducted this retrospective population-based cohort study to determine 1) pregnancy outcomes of women with OAPS and TAPS, 2) association between aPL profiles and specific obstetric complications, and 3) long-term thromboembolic and obstetric morbidity.

## 2 Methods

### 2.1 Study population

This population-based retrospective cohort study was conducted at the Soroka University Medical Center (SUMC). The Department of Obstetrics and Gynecology attended in recent years about 17,000 deliveries annually and established a maternity database of 320,000 deliveries occurring between 1998 and 2020. The study included all women with a singleton gestation who delivered at the SUMC between the years 1998 and 2020. Women were excluded if the fetus had congenital structural and/or chromosomal abnormalities.

Demographic data included maternal age at delivery, ethnicity, gravidity, and parity. Clinical characteristics included systemic lupus erythematosus (SLE), smoking, chronic hypertension, and hypercholesterolemia. Thrombotic complications included hospitalizations after the first diagnosis with any of the following diagnoses: deep vein thrombosis, pulmonary embolism (PE), stroke, transient ischemic attack (TIA), or coronary artery disease. APS-related treatments, including aspirin, enoxaparin, hydroxychloroquine, and steroids at any time throughout the study period, either during hospitalization or temporary\chronic use after discharge, were recorded. Both OAPS and TAPS patients received LMWH and aspirin during pregnancy, whereas long-term treatment with warfarin was administered only to patients diagnosed with TAPS.

The SUMC institutional review board approved the study (#0157-20-SOR).

### 2.2 Clinical definitions

APS was defined as the combination of arterial or venous thrombosis and/or pregnancy complications, characterized by fetal loss after the 10th week of gestation, recurrent early miscarriages, intrauterine growth restriction, or severe preeclampsia, with 1 or more of the following: 1) positive lupus anticoagulant (LAC); 2) anticardiolipin (aCL) antibodies (immunoglobulin [Ig]G or IgM); and/or 3) anti-β2-glycoprotein I (β2GPI) antibodies (IgG/IgM), with titer in the medium or high concentration (>40 units/mL IgG or IgM > 99th percentile) in 2 consecutive measurements at least 12 weeks apart [3]. Triple-positive APS profile was defined as positivity to LAC, aCL, and anti-β2GPI antibodies [8]. Tests were performed as a part of the workup for an obstetric complication or thrombotic event. All 3 tests were performed on all patients. The date of APS diagnosis was defined as the date of the first positive APS profile test. In patients with criteria for both OAPS and TAPS, groups were assigned according to their primary phenotypic presentation.

### 2.3 Outcomes

Long-term outcomes included complications that occurred after the diagnosis of APS**.** Obstetric complications included preterm birth, fetal growth restriction (FGR), preeclampsia, fetal loss, placental abruption, and stillbirth. Early preterm birth was defined as a birth of a morphologically normal neonate before the 34th week of gestation. FGR was defined as estimated fetal weight <10th percentile for gestational age [9]. Preeclampsia was defined as new onset hypertension and proteinuria or hypertension and significant end-organ dysfunction occurring after 20 weeks of gestation or postpartum [10]. Placental abruption was defined as the separation of a normally implanted placenta before delivery, diagnosed clinically during delivery or at cesarean section. Stillbirth was defined as the loss of a fetus at or after 20 weeks of pregnancy. Late fetal loss refers to a spontaneous intrauterine death of a fetus after the 10th week of gestation.

Neonatal outcomes included gestational age at delivery, birthweight, Apgar score at 1 and 5 minutes, and small for gestational age (SGA) neonate [11]. Gestational age at delivery was defined as the last menstrual period or by ultrasound examination during the first trimester. Low Apgar scores indicate scores below 7, and SGA is defined as a birth weight below the 10th percentile for gestational age**.**

### 2.4 Statistical analysis

When appropriate, univariate comparisons for dichotomic variables used the chi-squared or Fisher's exact test, and Student's *t*-test or Mann–Whitney U-test was used for quantitative variables. We performed generalized linear regression models for multivariate analysis of continuous variables and generalized estimating equation models for multivariate analysis of dichotomic variables. A considerable proportion of women included in our dataset have more than 1 entry due to multiple pregnancies. We used generalized estimating equation models to account for clustering (every woman constituted a cluster) and adjusted the models for maternal age, gravidity, and ethnicity. Long-term outcomes of patients were assessed by logistic regression. We used IBM SPSS software version 25.0 (IBM, SPSS Inc) for statistical analysis.

## 3 Results

**Figure 1:**
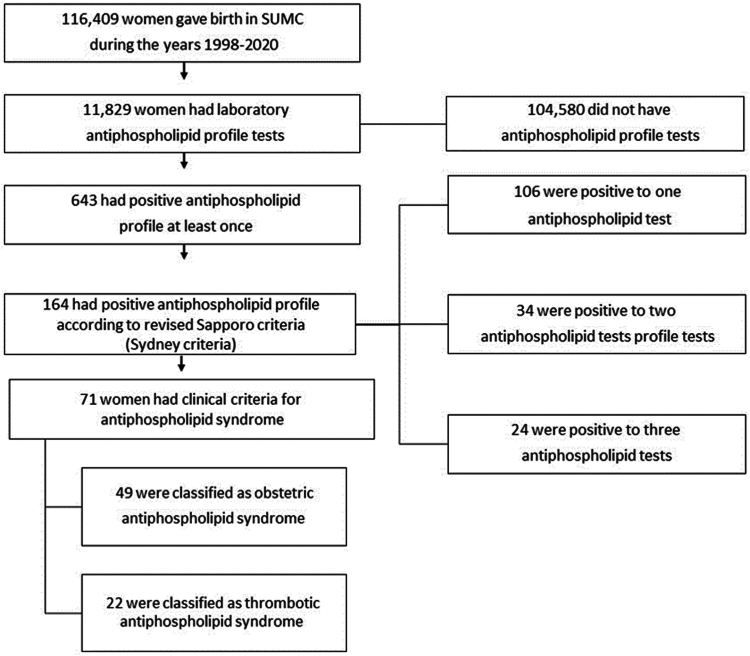
Study population. A descriptive distribution of individuals within the study population who met the predefined inclusion criteria, as well as the prevalence and classification of antiphospholipid syndrome within the study population. SUMC, Soroka University Medical Center.

A total of 116,409 women with 320,455 pregnancies met the inclusion criteria. Among them, 10.16% (11,829/116,409) underwent laboratory antiphospholipid profile testing (Figure 1). In the study population, 0.5% (643/116,409) of women tested positive for the antiphospholipid profile at least once, whereas 0.14% (164/116,409) met the laboratory criteria for APS. Among women with a positive laboratory profile for APS, 64.63% (106/164) tested positive for 1 test, 20.73% (34/164) tested positive for 2 tests, and 2.43% (4/164) tested positive for all 3 tests. Of all patients, 0.06% (71/116,409) met both laboratory and clinical criteria for APS diagnosis. Based on the clinical manifestations at the time of diagnosis, 69.01% (49/71) were classified as OAPS and 30.9% (22/71) as TAPS.

Table 1 outlines the demographics and clinical characteristics of the study groups. OAPS displayed higher incidences of chronic hypertension and SLE compared with the general obstetric population, while TAPS patients showed elevated rates of SLE, chronic hypertension, and hypercholesterolemia. SLE rates were greater in TAPS (31.82%) compared with OAPS (14.29%), though this disparity did not reach statistical significance (*P* = .09).

**Table 1:** Demographic and clinical characteristics of patients with antiphospholipid syndrome.

Variable		General obstetric population (*N* = 116,338)	TAPS (*n* = 22)	OAPS (*N* = 49)	*P* value (TAPS vs general obstetric population)	*P* value (OAPS vs general obstetric population)	*P* value (TAPS vs OAPS)
Ethnicity	Bedouins	47,219 (40.6%)	5 (22.7%)	16 (32.7%)	.12	.31	.58
	Jewish	69,119 (59.4%)	17 (77.3%)	33 (67.3%)			
Gravidity, median		4 (2-6)	2.5 (1-4.25)	5 (3-7)	-	-	.045
Parity, median		3 (2-5)	2 (1-3)	3 (1-4)	-	-	.52
Maternal age at delivery (y)^a^		29.02 ± 0.01	30.8 ± 1.1	28.72 ± 0.5	-	-	.07
SLE		179 (0.2%)	7 (31.8%)	7 (14.3%)	**<.001**	**<.001**	.09
Smoking		3786 (3.3%)	1 (4.6%)	6 (12.2%)	.51	.21	.79
Chronic hypertension		4923 (4.2%)	8 (36.4%)	6 (12.2%)	**<.001**	**<.01**	**.02**
Hypercholesterolemia		3272 (2.8%)	6 (27.3%)	2 (4.1%)	**<.001**	.4	**.004**

Data are presented as *n* (%), mean ± SD, or median (IQR).

Bolded values are the statistically significant findings.

OAPS, obstetric antiphospholipid syndrome; SLE, systemic lupus erythematosus; TAPS, thrombotic antiphospholipid syndrome.

a The mean maternal age in each group of patients was analyzed according to the total number of deliveries in each group throughout the study period. TAPS had 38 deliveries, OAPS had 145 deliveries, and patients of the general obstetric population had 320,455 deliveries.

**Figure 2:**
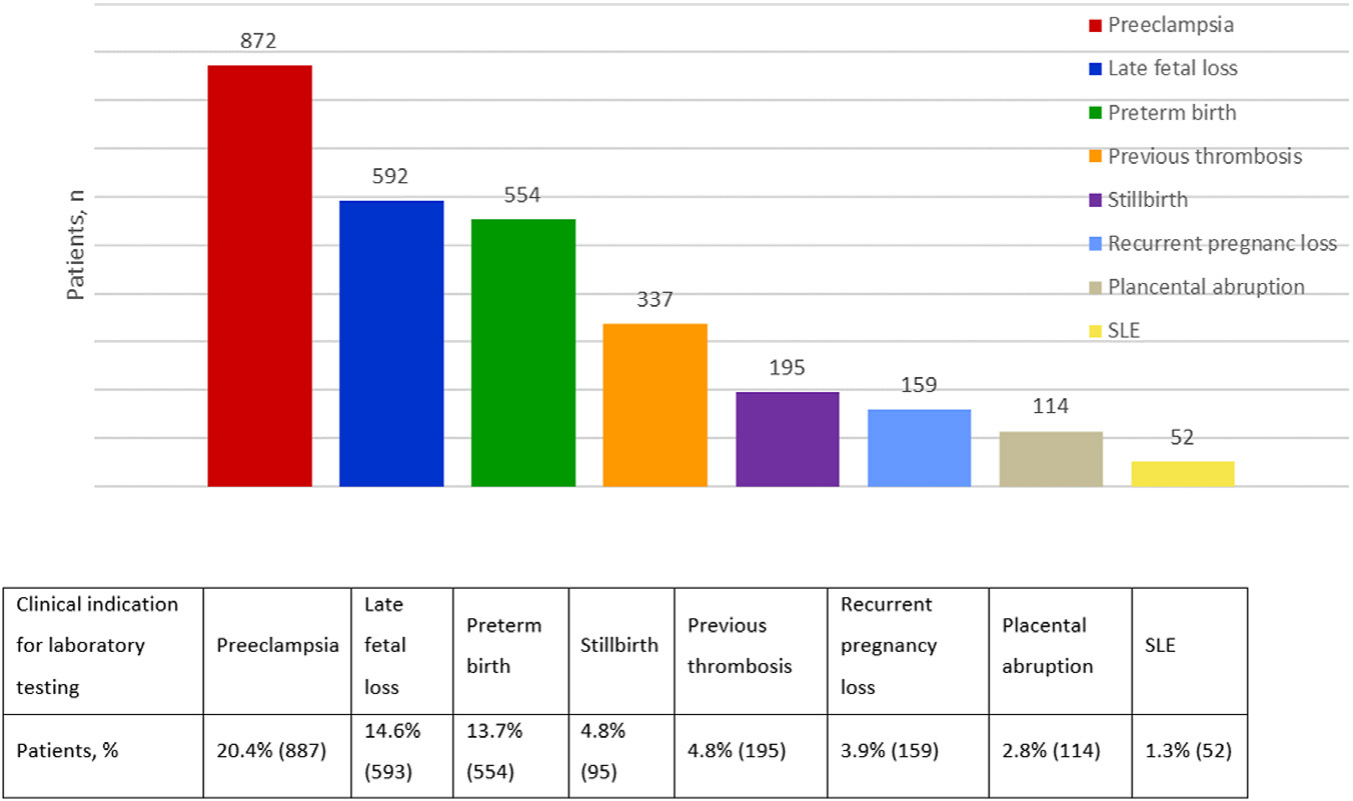
Clinical indications for antiphospholipid antibody testing (*n* = 4055). The distribution of patients was categorized according to their clinical indications for laboratory antiphospholipid antibody testing at the time of initial diagnosis of antiphospholipid syndrome. SLE, systemic lupus erythematosus.

Figure 2 depicts the clinical indications for laboratory testing of aPLs. Among 4055 patients, at least 1 clinical indication was identified. Of these, 33.36% (1445/4055) had multiple indications, with 23.75% (963/4055) presenting 2 clinical indications and 5.45% (221/4055) demonstrating 3 indications. Preeclampsia accounted for the most common indication, at 20.39% (827/4055).

Supplementary Figure S1 illustrates aPL profiles at the time of the first diagnosis in women with OAPS or TAPS. In both groups, the most prevalent positive tests were LAC (OAPS: 85.7% [42/49]; TAPS: 86.4% [19/22]), followed by aCL IgG (OAPS: 32.65% [16/49]; TAPS: 40.9% [9/22]) and anti-β2GPI IgG (18.4% [9/49] in OAPS; 22.7% [5/22] in TAPS). Upon diagnosis, OAPS demonstrated a higher rate of anti-β2GPI IgM compared with TAPS.

Supplementary Figure S2 describes the distribution of treatment among patients diagnosed with either OAPS or TAPS throughout the study period. The most prevalent treatments were LMWH (73.47% [36/49] in OAPS and 54.55% [12/22] in TAPS) and aspirin (48.98% [24/49] in OAPS and 54.55% [12/22] in TAPS). Nevertheless, we do not have data regarding the dosage of LMWH or whether LMWH was used in a prophylactic or therapeutic dose. In OAPS, a higher percentage of patients were treated with steroids (38.78% [19/49]) compared with hydroxychloroquine (20.41% [10/49]).

Table 2 presents obstetric morbidity prior to the APS diagnosis. When stratified according to APS phenotypes, both groups displayed higher rates of preeclampsia compared with the general obstetric population (6/22 [27.3%] in TAPS, 17/49 [34.7%] in OAPS, and 12,171/116,338 [10.5%] in the general obstetric population). Compared with TAPS, OAPS patients had higher rates of history of preterm birth and FGR. Despite the differences in the rates of preeclampsia between TAPS and OAPS, the difference did not reach statistical significance (*P* = .05).

**Table 2:** Obstetric complications before the diagnosis of antiphospholipid syndrome.

Variable	General obstetric population (*N* = 116,338)	TAPS (*n* = 22)	OAPS (*n* = 49)	*P* value (APS vs general obstetric population)	*P* value (TAPS vs general obstetric population)	*P* value (OAPS vs general obstetric population)	*P* value (OAPS vs TAPS)
History of FGR	17,411 (15.0%)	3 (13.6%)	21 (42.9%)	**<.001**	.86	**<.001**	**.02**
History of preterm birth	22,287 (19.2%)	4 (18.2%)	30 (61.2%)	**<.001**	.91	**<.001**	**.001**
History of preeclampsia	12,171 (10.5%)	6 (27.3%)	17 (34.7%)	**<.001**	**.01**	**<.001**	.54
History of late fetal loss	4412 (3.8%)	0 (0%)	30 (61.2%)	**<.001**	-	**<.001**	-
History of stillbirth	1544 (1.3%)	1 (4.6%)	7 (14.3%)	**<.001**	.18	**<.001**	.23
History of placental abruption	2252 (1.9%)	1 (4.6%)	3 (6.1%)	**.02**	.37	**.03**	.79

Data are presented as *n* (%), mean ± SD, or median (IQR).

Bolded values are the statistically significant findings.

APS, antiphospholipid syndrome; FGR, fetal growth restriction; OAPS, obstetric antiphospholipid syndrome; TAPS, thrombotic antiphospholipid syndrome.

Table 3 summarizes neonatal characteristics across the study period, including pregnancies without anticoagulant treatment. OAPS neonates exhibited higher rates of low Apgar scores at 1 and 5 minutes compared with the general obstetric population, with no discernible differences in Apgar scores observed between TAPS neonates and the general obstetric population. Both TAPS and OAPS had lower mean birthweight and shorter mean gestational age compared with women without a diagnosis of APS.

**Table 3:** Neonatal characteristics in thrombotic and obstetric antiphospholipid syndrome.

Variable	General obstetric population (*N* = 116,338)	TAPS (*n* = 22)	OAPS (*n* = 49)	*P* value (TAPS vs general obstetric population)	*P* value (OAPS vs general obstetric population)	*P* value (TAPS vs OAPS)
Mean birthweight (g)	3154.6 ± 500.9	2849.7 ± 844.7	2428.9 ± 962.0	**.004**	**<.001**	.08
Gestational age (wk)	38.9 ± 2.0	37.6 ± 3.9	35.4± 4.7	**<.001**	**<.001**	.06
Low Apgar`1	2105 (1.8%)	2 (9.1%)	5 (10.2%)	.059	**<.001**	.88
Low Apgar`5	339 (0.3%)	0 (0%)	2 (4.1%)	1.0	**<.001**	.34

Data are presented as *n* (%), mean ± SD, or median (IQR).

Bolded values are the statistically significant findings.

OAPS, obstetric antiphospholipid syndrome; TAPS, thrombotic antiphospholipid syndrome.

### 3.1 Adverse obstetric and thrombotic outcomes under standard treatment protocols

Table 4 describes the long-term thrombotic outcomes in patients with APS. TAPS patients undergoing long-term treatment with warfarin exhibited a higher incidence of stroke/TIA and PE compared with OAPS patients. Of note, patients in the OAPS group did not receive anticoagulant treatment beyond the postpartum period (stroke/TIA: 18.18% [4/22] in TAPS and 2.04% [1/49] in OAPS; PE: 13.64% [3/22] in TAPS and 2.04% [1/49] in OAPS). Women with OAPS had an unadjusted increased lifetime risk for thrombotic complications in comparison with the general obstetric population (odds ratio [OR], 18.0; 95% CI, 8.7-37.2).

**Table 4:** Long-term obstetric and thrombotic outcomes in antiphospholipid syndrome patients.

Variable	TAPS (*n* = 22)	OAPS (*n* = 49)	*P* value (TAPS vs OAPS)
Stroke/TIA	4 (18.2%)	1 (2.0%)	**.01**
PE	3 (13.6%)	1 (2.0%)	**.05**
CAD	2 (9.1%)	1 (2.0%)	.17
DVT	5 (22.7%)	6 (12.2%)	.26
Late fetal loss	0 (0%)	10 (20.4%)	**.02**
Placental abruption	1 (4.6%)	0 (0%)	.13
Preeclampsia	2 (9.1%)	7 (14.3%)	.54
FGR	1 (4.6)	7 (14.3%)	.23
Preterm birth	2 (9.1%)	12 (24.5%)	.13
SGA	1 (4.6%)	4 (8.2%)	.58
Stillbirth	1 (4.6%)	2 (4.1%)	.93

Data are presented as *n* (%), mean ± SD, or median (IQR).

Bolded values are the statistically significant findings.

CAD, coronary artery disease; DVT, deep vein thrombosis; FGR, fetal growth restriction; OAPS, obstetric antiphospholipid syndrome; PE, pulmonary embolism; SGA, small for gestational age; TAPS, thrombotic antiphospholipid syndrome; TIA, transient ischemic attack.

A comparison of obstetric outcomes between women with TAPS and OAPS showed higher rates of late fetal loss in OAPS at 20.41% (10/49) compared with 0% in TAPS (Table 4). When categorized by the number of pregnancies, no significant difference was found between the groups regarding late fetal loss, placental abruption, preeclampsia, FGR**,** preterm birth, SGA, and stillbirth (Table 5).

**Table 5:** Long-term obstetric outcomes in antiphospholipid syndrome patients by the number of pregnancies under antithrombotic treatment.

Variable	TAPS (*n* = 9)	OAPS (*n* = 72)	*P* value (TAPS vs OAPS)
Late fetal loss	0 (0%)	4 (5.6%)	1.0
Placental abruption	1 (11.1%)	0 (0%)	.11
Preeclampsia	2 (22.2%)	7 (9.7%)	.27
FGR	2 (22.2%)	7 (9.7%)	.27
Preterm birth	2 (22.2%)	14 (19.4%)	.45
SGA	1 (11.1%)	4 (5.6%)	.45
Stillbirth	0 (0%)	2 (2.8%)	1.0

Data are presented as *n* (%), mean ± SD, or median (IQR).

FGR, fetal growth restriction; OAPS, obstetric antiphospholipid syndrome; SGA, small for gestational age; TAPS, thrombotic antiphospholipid syndrome.

Table 5 compares obstetric complications in OAPS and TAPS patients following the initial APS diagnosis and under antithrombotic treatment to the general obstetric population, adjusting for maternal age, gravidity, and ethnicity. OAPS patients had elevated risks for fetal loss (adjusted OR [aOR], 15.3; 95% CI, 8.5-27.5), FGR (aOR, 4.3; 95% CI, 2.6-6.9), stillbirth (aOR, 5.9; 95% CI, 2.2-15.4), preterm birth (aOR, 4.5; 95% CI, 2.8-7.1), and placental abruption (OR, 4.8; 95% CI, 1.5-15.3) compared with TAPS. TAPS patients had an increased risk only for preeclampsia (OR, 3.1; 95% CI, 1.2-8) compared with OAPS.

Table 6 compares obstetric complications and neonatal outcomes between OAPS and TAPS patients and the general obstetric population after the initial APS diagnosis and under antithrombotic treatment, adjusted for maternal age, gravidity, and ethnicity. OAPS patients displayed a lower mean birthweight of 564.2 g (aOR, 564.2; 95% CI, −792.4 to −336.0), shorter gestational age at delivery of 17.3 days (aOR, 17.5; 95% CI, −23.8 to −11.2), increased rates of SGA (aOR, 4.0; 95% CI, 2.4-6.6), and lower Apgar'1 (aOR, 2.6; 95% CI, 1.5-4.4) and Apgar'5 (aOR, 3.7; 95% CI, 1.3-10.4). TAPS patients did not exhibit an increased risk for adverse neonatal outcomes.

**Table 6:** Comparison of obstetric complications and neonatal outcomes in obstetric and thrombotic antiphospholipid syndrome patients after the initial antiphospholipid syndrome diagnosis and under antithrombotic treatment compared with the general obstetric population, adjusted for maternal age, gravidity, and ethnicity.

Variable	OAPS		TAPS	
	Unadjusted odds ratio	Adjusted odds ratio	Unadjusted odds ratio	Adjusted odds ratio
Late fetal loss	40.1 (22.5-71.2)	**15.3 (8.5-27.5)**	-	-
FGR	3.9 (2.4-6.4)	**4.3 (2.6-6.9)**	1.4 (0.3-6.4)	1.6 (0.4-6.9)
Preeclampsia	4.1 (2.4-7.2)	**4.4 (2.5-7.7)**	3.6 (1.4-9.4)	**3.1 (1.2-8)**
Stillbirth	12.4 (5.6-27.6)	**5.9 (2.2-15.4)**	3.5 (0.5-26.3)	4.1 (0.5-33.6)
Preterm birth	4.3 (2.7-6.9)	**4.5 (2.8-7.1)**	1.1 (0.4-3.2)	1.1 (0.4-3.1)
Placental abruption	4.8 (1.5-15.3)	**4.8 (1.5-15.3)**	4.4 (0.6-31.6)	4.4 (0.6-32.3)
Thrombotic events^a^	**18.0 (8.7-37.2)**	-	-	-
SGA	3.7 (2.3-5.9)	**4.0 (2.4-6.6)**	1.3 (0.3-5.3)	1.4 (0.4-5.6)
Low Apgar'1	2.6 (1.5-4.4)	**2.6 (1.5-4.4)**	1.9 (0.6-6.2)	2.0 (0.4-9.0)
Low Apgar'5	3.4 (1.3-9.2)	**3.7 (1.3-10.4)**	3.2 (0.4-23.6)	3.6 (0.5-25.8)
Mean birthweight (g)	−545 (−638.5 to −451.4)	−564.2 (−794.2 to −336.0)	−235.2 (−417.9 to −52.5)	−251.9 (−562.2 to 58.4)
Gestational age (d)	−17.3 (−20 to 14.6)	−17.5 (−23.8 to −11.2)	−6.8 (−12.2 to −1.5)	−6.5 (−16.1 to 3.0)

Data are presented as *n* (%), mean ± SD, or median (IQR).

Bolded values are the statistically significant findings.

FGR, fetal growth restriction; OAPS, obstetric antiphospholipid syndrome; SGA, small for gestational age; TAPS, thrombotic antiphospholipid syndrome.

a All events that are part of the clinical criteria of TAPS throughout the entire study period.

### 3.2 Thrombosis in APS patients diagnosed with triple-positive laboratory profile

Supplementary Table describes long-term obstetric and thrombotic outcomes for APS patients with and without triple-positive antiphospholipid profile. Triple-positive APS patients had a higher rate of deep vein thrombosis, stroke/TIA, and obstetric complications than those with non–triple-positive APS. There was no difference in the rate of obstetric complications between triple-positive APS patients and non–triple-positive APS.

## 4 Discussion

### 4.1 Principal findings of the study

OAPS patients showed a higher rate of placenta-related complications and neonatal morbidity compared with both TAPS patients and those without an APS diagnosis, despite the administration of antithrombotic treatment during gestation and postpartum. TAPS patients exhibited an elevated rate of preeclampsia, with no significant differences noted in other obstetric complications. Patients initially diagnosed with OAPS had a significantly elevated risk of thrombotic events compared with the general obstetric population.

### 4.2 Results in the context of what is known

Preeclampsia emerged as the predominant obstetric complication in both the thrombotic and obstetric phenotypes, occurring in pregnancies preceding the initial diagnosis of APS and persisting in subsequent pregnancies despite standard prophylactic treatment. In a retrospective cohort study by Bramham et al. [12], although not statistically significant, TAPS patients had a higher incidence of preeclampsia than OAPS patients. The study was conducted at a tertiary lupus clinic, and TAPS patients demonstrated notably higher rates of SLE compared with the OAPS group. Given the association between SLE in APS and preeclampsia [13], this correlation could potentially contribute to the incidence observed in patients with TAPS. OAPS patients also had high rate of neonatal complications, with and without prophylactic treatment. Similarly, Erton et al. [14] showed that OAPS patients had higher rate of lower birth weight compared with TAPS. Earlier studies found no differences in rates of stillbirth, FGR, and late fetal loss [15] among pregnant women with TAPS and OAPS.

OAPS patients in our cohort showed higher anti-β2GPI IgM prevalence than TAPS at time of diagnosis. Positivity to anti-β2GPI is associated with the high incidence of obstetric complications [16]; however, few studies have compared IgM and IgG isotypes between TAPS and OAPS.

Jiang et al. [17] noted an increased anti-β2GPI positivity in OAPS patients without distinguishing between IgG and IgM isotypes. In a study by Anunciación-Llunell et al. [18], OAPS patients also exhibited higher positivity for aβ2GPI IgM compared with TAPS patients. These different laboratory profiles could be linked to the difference in obstetric and neonatal outcomes in APS phenotypes.

TAPS patients (under long-term anticoagulation) exhibited a higher rate of recurrent thrombosis compared with individuals without APS. Additionally, OAPS patients, who usually do not receive anticoagulation outside of pregnancy, faced a heightened risk of thromboembolic events. Several studies reported a high risk of thrombosis in OAPS patients, yet these results were not compared with TAPS patients. [19,20]. The European Registry on Obstetric Antiphospholipid Syndrome study [21,22] reported a low incidence of thromboembolic events in OAPS patients, but it did not compare this with the general obstetric population or individuals diagnosed with TAPS. Rottenstreich et al. [23] found that women diagnosed with either the TAPS or OAPS phenotype were prone to thromboembolic complications. Their cohort included a substantial number of APS patients with a triple-positive laboratory profile, linked to adverse APS outcomes [1,14] and possibly contributing to the observed heightened rate of APS-related complications. In a study by Cervera [6], TAPS patients showed elevated recurrent thrombosis rates, with 23% of the cohort not receiving antithrombotic treatment during the study period. Nonadherence to long-term treatment in APS patients has been widely described [24], and this could be an important factor in recurrent events.

The population in our cohort was split into Jewish and Arab Bedouin. The Jewish population is more urban and settled, while the Bedouins are nomadic in nature and mostly live in a rural setting, have a high rate of consanguinity marriage and high number of children per family, and practice low rate of contraceptives. These factors have a substantial contribution to the differences between the 2 populations, as compliance with treatment and regular prenatal follow-up are lower in the Bedouin population. Due to these differences, we have adjusted all our results to ethnicity in the multivariate analyses.

Our cohort includes patients with both TAPS and OAPS. Most previous published studies either excluded patients with TAPS, did not make the distinction between the 2 entities, or reported their results for both groups combined. Nevertheless, our cohort is very similar to previously published cohorts [15,25,26] with regard to chronic disease and risk factors. In contrast, Tao et al. [27] described a retrospective cohort in which aCL and anti-ß2GP1 were positive if antibody titers for IgG and/or IgM isotypes were ≥20; some of the patients in this cohort would not meet APS diagnostic criteria. Compared to this cohort, more patients in our cohort had concurrent SLE, and more patients had a history of thrombosis, which is in agreement with strict inclusion only of patients who were diagnosed with APS according to the criteria. We also had more patients of Caucasian ethnicity in comparison with this study. The PROMMISE (Predictors of pRegnancy Outcome: bioMarkers In antipho-spholipid antibody Syndrome and Systemiclupus Erythematosus) study [28] described a cohort with less gravidity. Hence, we believe that our findings are generalizable and novel with regard to the separate description of obstetric and thrombotic outcomes in TAPS and OAPS patients.

### 4.3 Clinical implications

Our findings suggest that extended antithrombotic treatment should be considered for OAPS patients due to their risk of thromboembolic sequelae. An evaluation by Erkan et al. [29] demonstrated that acetylsalicylic acid effectively served as thrombosis prophylaxis in OAPS patients. A meta-analysis supports the protective role of low-dose aspirin for primary prophylaxis against thrombosis, especially in asymptomatic aPL-positive individuals and those with SLE or OAPS [24].

Pregnancies in OAPS patients, both before and after their diagnosis, exhibited neonatal complications linked to early-onset preeclampsia [30,31]. Women with TAPS showed a notable prevalence of chronic hypertension and hypercholesterolemia, resembling mechanisms typically linked to late-onset preeclampsia [32]. Recent research [33] revealed a link between an imbalance in angiogenic factors and early-onset preeclampsia, characterized by elevated levels of soluble fms-like tyrosine kinase-1 and decreased placental growth factor. Abnormal PlGF-to-sFlt-1 ratio holds negative predictive value for adverse obstetric and neonatal outcomes in APS [[34], [35], [36]]. These findings support the investigation of angiogenesis-related biomarkers as a viable approach to detecting early-onset preeclampsia in patients with OAPS [37].

Our results support the notion previously suggested [7,17] that IgG antibodies from patients with either pure TAPS or OAPS can evoke distinct biological effects. It has been proposed that, unlike the vascular endothelium, the high expression of β2GP1 in decidual endothelial cells may predispose it to the proinflammatory response triggered by anti-β2GP1 [38,39]. Indeed, in a study by Poulton et al. [40], IgG antibodies impede trophoblast invasion in OAPS patients with multiple miscarriages and not in those with TAPS. Additionally, examination of placentas from OAPS patients exhibited inflammation, whereas this was absent in cases of TAPS [41].

### 4.4 Research implications

We present in our study that despite the administration of antithrombotic treatments, a substantial proportion of women with TAPS and OAPS develop pregnancy complications. While in OAPS, we move to second and third lines of treatment, such as hydroxychloroquine, steroids, and intravenous immunoglobulins, TAPS patients may benefit from the addition of statins. TAPS patients had higher rates of cardiovascular morbidity compared with OAPS. Previous research emphasizes that cardiovascular morbidity is involved in the thrombotic phenotype mechanism [42,43], and recent research [43] focuses on simvastatin and fluvastatin in the treatment of APS. Simvastatin and fluvastatin hinder endothelial activation linked to anti-β2GPI. Fluvastatin has the potential to mitigate tissue factor expression induced by antiphospholipids in endothelial cells. APS patients treated with fluvastatin also showed reductions in inflammatory and prothrombotic biomarkers [44].

In our cohort, triple-positive APS patients exhibited an increased risk for long-term thromboembolic events compared with non–triple-positive patients, even with permanent anticoagulation in those diagnosed based on a thrombotic event, with no significant rise in obstetric complications. These results further support the need for specific treatment in different APS phenotypes. Recently, a notable emphasis on neutrophil extracellular trap [45,46] formation has been observed in TAPS, particularly in individuals with triple antiphospholipid positivity and recurrent thrombosis [43]. Potential therapeutic strategies include DNase-mediated digestion of neutrophil extracellular traps and anti-β2GPI-induced tumor necrosis factor-α effects through adalimumab and certolizumab [47]. Further clinical research is needed to stratify specific treatments in this high-risk population.

### 4.5 Strengths and limitations

The strength of this study derives from the large cohort and long follow-up. This is the first study of this size that compares clinical and laboratory characteristics of thrombotic and OAPS patients. SUMC, being the sole tertiary hospital in the south of Israel, likely admitted women from our study cohort for subsequent deliveries and treatment of thromboembolic complications. Limitations include the retrospective nature that prevents us from proving causality. We referenced prior research to strengthen the connection between potential causal factors and pregnancy outcomes in APS patients.

Secondly, there is a lack of documentation regarding drug dosage and therapy adherence. Nevertheless, treatment for APS at SUMC aligns with international consensus guidelines, and it is presumed that the administered treatment adhered to standard international protocols. Another limitation is the modest size of the patient cohort, yet it aligns with APS prevalence in the general population.

## 5 Conclusions

Our study emphasizes that pregnancies in OAPS patients, despite receiving similar treatment in both groups, exhibited inferior obstetric and neonatal outcomes compared with TAPS patients. Women initially diagnosed with the obstetric phenotype have considerable risk to develop long-term thromboembolic events; therefore, prolonged treatment should be considered in these patients. Nevertheless, the latter observation requires future studies that are targeted to address this question. Further clinical research is warranted to facilitate tailored treatment approaches in specific APS subgroups.
