# Supplementary material for: Obstetric antiphospholipid syndrome carries an increased lifetime risk for obstetric and thrombotic complications—a population-based study
Source: Res Pract Thromb Haemost. 2024 Apr 29;8(4):102430. doi: 10.1016/j.rpth.2024.102430 (PMC11127162; doi:10.1016/j.rpth.2024.102430)
Supplement: Supplementary Figures and Table [file mmc1.docx]

**Supplementary Material**

Content:

Figure S1- **The distribution of positive antiphospholipid tests**

Figure S2- **Treatment distribution in antiphospholipid patients**

Supplementary Table 1- **Long-term obstetric and thrombotic outcomes for antiphospholipid syndrome patients with and without triple positive antiphospholipid profile**

**Figure S1 – The distribution of positive antiphospholipid tests**

The distribution of positive antiphospholipid laboratory tests in women diagnosed with obstetric or thrombotic antiphospholipid syndrome, based on the clinical presentation at the time of their initial diagnosis

OAPS: Obstetric antiphospholipid syndrome; TAPS: Thrombotic antiphospholipid syndrome; LAC: Lupus anticoagulant; aCL: anticardiolipin; anti-β2GPI: anti-β2-glycoprotein I.


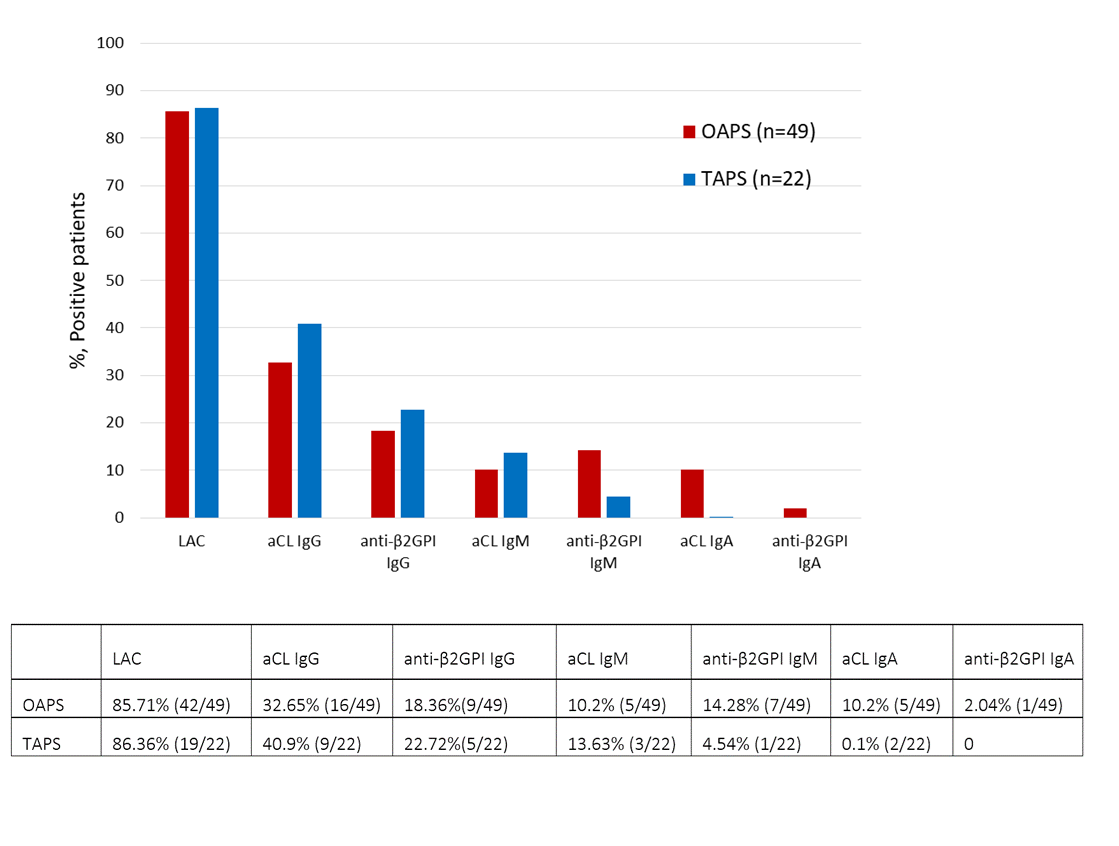


**Figure S2 – Treatment distribution in antiphospholipid patients**

The distribution of treatment among patients diagnosed with either obstetric or thrombotic antiphospholipid syndrome throughout the study period.

OAPS: Obstetric antiphospholipid syndrome; TAPS: Thrombotic antiphospholipid syndrome; LMWH: Low molecular weight heparin.


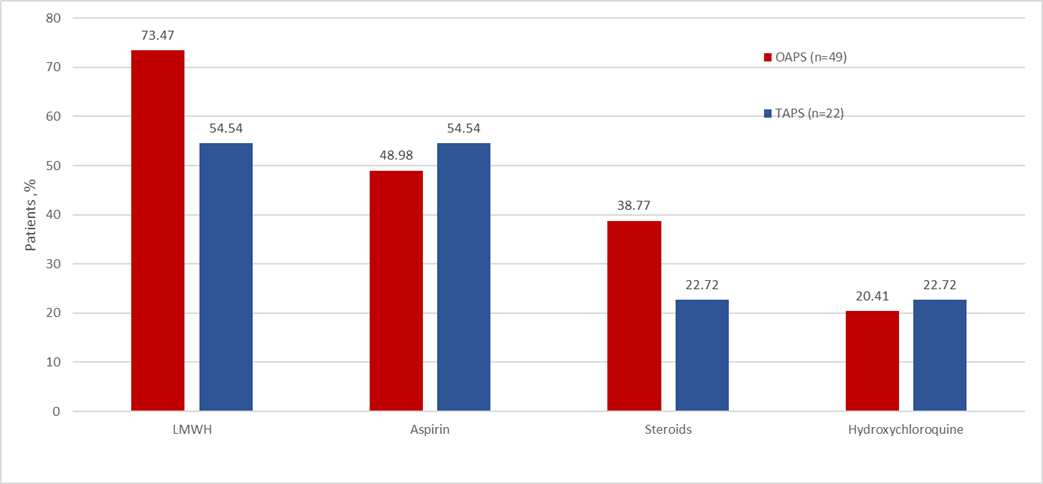


**Supplementary table 1– Long-term obstetric and thrombotic outcomes for antiphospholipid syndrome patients with and without triple positive antiphospholipid profile**

| **P-Value** | **Non-triple positive**  **APS profile**** **(n=57)** | **Triple positive**  **APS profile* (n=14)** | **Variable** |
| --- | --- | --- | --- |
| **0.005** | 5  (8.8%) | 6  (42.9%) | DVT |
| **0.049** | 2  (3.5%) | 3  (21.4%) | Stroke/TIA |
| 0.17 | 2  (3.5%) | 2  (14.3%) | PE |
| 1.0 | 3  (5.3%) | 0  (0%) | CAD |
| 1.0 | 7  (12.3%) | 2  (14.3%) | Preeclampsia |
| 0.65 | 6  (10.5%) | 2  (14.3%) | FGR |
| 0.197 | 0  (0%) | 1  (7.1%) | Placental abruption |
| 0.45 | 10  (17.5%) | 4  (28.6%) | Preterm birth |
| 0.25 | 3  (5.3%) | 2  (14.3%) | SGA |
| 0.09 | 1  (1.8%) | 2  (14.3%) | Stillbirth |
| 1.0 | 8  (14.0%) | 2  (14.3%) | Late fetal loss |

*Data presented as Number (%), mean ± standard deviation or median and (interquartile range – IQR)

*****Triple-positive antiphospholipid syndrome was defined as positivity to LAC, aCL, and anti-β2GPI antibodies, on two or more occasions at least 12 weeks apart, with the clinical criteria of APS, fulfilling the revised Sapporo criteria.

** Non-triple positive APS was defined as a positive aPL profile to one or two aPL antibodies according to Sapporo criteria on two or more occasions, at least 12 weeks apart, and clinical criteria of APS, fulfilling the revised Sapporo criteria.

OAPS: Obstetric antiphospholipid syndrome; TAPS: Thrombotic antiphospholipid syndrome; FGR: fetal growth restriction; SGA: small for gestational age; DVT: deep vein thrombosis; PE: pulmonary embolism; TIA: transient ischemic attack.
